# Interfacial reconstruction in La_0.7_Sr_0.3_MnO_3_ thin films: giant low-field magnetoresistance†

**DOI:** 10.1039/d0na00287a

**Published:** 2020-05-07

**Authors:** Umesh Kumar Sinha, Bibekananda Das, Prahallad Padhan

**Affiliations:** Department of Physics, Indian Institute of Technology Madras Chennai 600036 India Padhan@iitm.ac.in

## Abstract

Herein, interfacial reconstruction in a series of La_0.7_Sr_0.3_MnO_3_ (LSMO) films grown on a (001) oriented LaAlO_3_ (LAO) substrate using the pulsed plasma sputtering technique is demonstrated. X-ray diffraction studies suggested that the LSMO film on LAO was stabilized in a tetragonal structure, which was relaxed in-plane and strained along the out-of-plane direction. The interfacial reconstruction of the LSMO–LAO interface due to the reorientation of the Mn ion spin induced spin-glass behavior due to the presence of non-collinear Mn ion spins. Consequently, the interface effect was observed on the Curie temperature, temperature-dependent resistivity, metal-to-semiconductor transition temperature, and magnetoresistance (MR). At a magnetic field of 7 T, MR decreased from 99.8% to 7.69% as the LSMO film thickness increased from 200 Å to 500 Å. A unique characteristic of the LSMO films is the large low-field MR after a decrease in the field from the maximum field. The observed temperature-dependent magnetization and low-temperature resistivity upturn of the LSMO films grown on LAO provide direct evidence that the low-field MR is due to the non-collinear interfacial spins of Mn. The present work demonstrates the great potential of interface and large low-field MR, which might advance the fundamental applications of orbital physics and spintronics.

## Abstract 2

The 200 Å thick La_0.7_Sr_0.3_MnO_3_ film grown on (001) oriented LaAlO_3_ at 300 mT field exhibits 60% MR, which decreases with an increase in the La_0.7_Sr_0.3_MnO_3_ film thickness due to a decrease in the non-collinear Mn ion spins near the interface.

## Graphical abstract

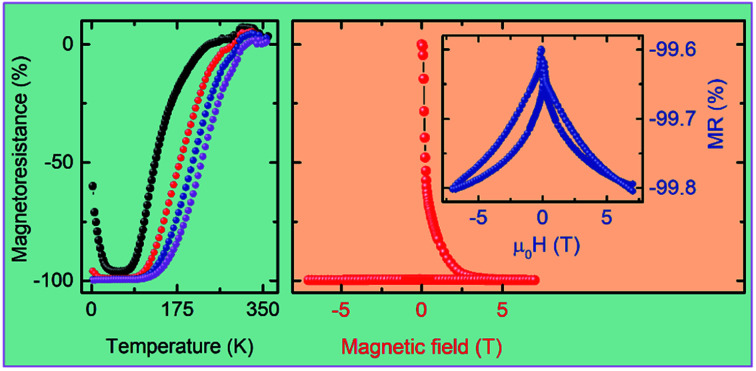

La_0.7_Sr_0.3_MnO_3_ (LSMO) exhibits metal-like electronic transport with striking properties such as ferromagnetic ordering at room temperature and nearly 100% spin polarization;^1,2^ it is often reported as the most itinerant electron material with the highest Curie temperature (*T*_C_ ∼ 369 K) among the known manganites.^1^ These unique properties make LSMO a fascinating material for technological applications.^3^ However, the growth of high-quality ultrathin LSMO films is very challenging because the bulk rhombohedral crystal structure of LSMO stabilizes in the form of a pseudocubic structure, which significantly modifies the bulk-like properties in LSMO films.^4–9^ Interestingly, structural reconstruction intermittently results in unique or improved functionalities in addition to control over the magnetic moment, *T*_C_^5,6^ and metal-to-insulator transition temperature (*T*_P_) of the LSMO films.^6–8^ The magnetic moment,^5^*T*_C_ and *T*_P_ of the LSMO films decrease as the LSMO film thickness decreases and below a certain film thickness, which is called the critical thickness, the metal-like conduction is suppressed by semiconducting behavior, where decoupling between ferromagnetism and metallicity occurs.^6–8^ The critical thickness of the LSMO films strongly depends on the substrate-induced stress.^8^ However, understanding the deviations in the physical properties of the films compared to that of bulk LSMO remains elusive. Nevertheless, many other factors have been found to account for this behavior, for example, the oxygen vacancies and charge transfer at the interfaces.

Extensive efforts are being devoted to exploring the electronic transport properties of LSMO films grown on a wide variety of substrates with or without a buffer layer.^8,9^ It has been demonstrated that the magnetoresistance (MR) behavior in LSMO films can be obtained at much lower magnetic fields by naturally or artificially modifying the Mn–O–Mn bond angle and bond length.^10^ Large low-field MR (LFMR) was observed by incorporating artificial grain boundaries in ferromagnetic manganite/spacer/ferromagnetic manganite trilayers. The highest LFMR value of 45–50% was obtained below a 0.02 T field at 4.2 K for La_0.67_Sr_0.33_MnO_3_/SrTiO_3_/La_0.67_Sr_0.33_MnO_3_ thin film junctions.^11,12^ The LFMR value of the composite films such as La_0.67_Sr_0.33_MnO_3_/ZnO was 23.9% at 0.5 T field and 10 K.^13^ The contribution of the grain boundary towards 23–27% LFMR at 0.02–0.1 T field and 77 K was explained by designing a single SrTiO_3_ bicrystal substrate grain boundary into an epitaxial film of La_0.7_Ca_0.3_MnO_3_.^14^ However, the growth of high-quality epitaxial manganite thin films with controlled and atomically smooth artificial grain boundaries is a very challenging task. Recently, we reported a unique pulsed plasma deposition (PPD) method, where the thin films of LSMO were stabilized epitaxially, even on the native SiO_2_ surface of the (001) oriented Si substrate by using a home-built RF magnetron sputtering system.^4,15^ Herein, we report the study of the spin dynamics in LSMO grown on an LaAlO_3_ (LAO) substrate, which exhibited giant LFMR (∼99%) at low temperatures. This LFMR strongly depends on the LSMO thickness (*t*_LSMO_), which suggests the interfacial origin of MR due to the variation in the bond length and bond angle at the interface.

The highly dense ceramic target of LSMO prepared by the solid-state reaction method was used to grow thin films on the (001) oriented LAO by adopting the PPD growth sputtering process. The sputtering system was designed in such a way that it (i) generates a horizontally orientated plasma, (ii) offers a larger cathode surface in front of the plasma, (iii) places the substrate heater inside the specified port, (iv) maintains a uniform temperature of the port wall using cooled water, and (v) provides an option to customize the target-to-substrate distance.^15^ The thin films were grown at 700  °C under 7 × 10^−3^ mbar of an argon and oxygen gas mixture. The chamber pressure was maintained by flowing the argon and oxygen gas at a 1 : 4 proportion during the deposition. The deposition was performed in pulsed mode by keeping the sputtering power density at 2.96 W cm^−2^. All the thin films were grown for different durations by altering the opening and closing of the shutter for a period of 10 s and 50 s, respectively. After the deposition of the desired thickness, the deposition chamber was filled with 50 mbar O_2_ followed by post-annealing for 45 min at 700  °C, and then, the film was cooled down to room temperature. LSMO was deposited on the (001) oriented LAO for six different growth periods. The LSMO films were found to be 100, 150, 200, 250, 375, and 500 Å thick, which was confirmed by optical measurements.

A four-cycle X-ray diffractometer was used to record the out-of-plane X-ray diffraction (XRD) patterns and reciprocal space mappings (RSMs) of the thin films. The temperature dependence of magnetization (*M*(*T*)) measurements for the out-of-plane orientation of the magnetic field were performed using a superconducting quantum interference device-based vibrating sample magnetometer. The electronic transport properties of the thin films were measured in the presence of a magnetic field using a physical property measurement system. The resistivity of the thin films was measured using the two-probe technique in the presence of an out-of-plane-oriented magnetic field. The temperature-dependent resistance of the films was measured while warming the films from the lowest temperature.

Fig. 1a shows the *θ*–2*θ* XRD patterns of the LSMO films with different thickness grown on (001) oriented LAO. The XRD patterns show only the (00*l*) oriented Bragg's peaks of LSMO and LAO without any signature of the other phase of LSMO. The out-of-plane pseudocubic lattice parameter (*c*_pc_) of the different LSMO films calculated from the (00*l*) peak positions of the XRD patterns is plotted in Fig. 1b. The *c*_pc_ of the LSMO films is independent of thickness and the average value is 3.99 Å, which is larger than the pseudo-cubic lattice parameter of the bulk LSMO (*c*_pc_ = 3.88 Å), as shown in Fig. 1.^16^ The larger *c*_pc_ value of the LSMO film compared to that of its bulk suggests that LAO provides out-of-plane tensile strain for the epitaxial growth of LSMO. The 100 Å thick LSMO film shows −2.68% tensile strain along the [001] orientation. As the LSMO film thickness increased to 150 Å, the strain also increased to −3.06%. However, the strain fluctuated between −2.65% to −3.06% as the LSMO film thickness varied from 100 to 500 Å [Fig. S2†]. Thus, the strain along [001] does not show any indication of relaxation with an increase in LSMO film thickness.

**Fig. 1:**
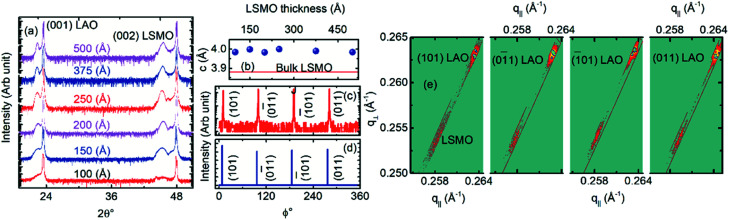
(a) *θ*–2*θ* X-ray diffraction patterns of the 100, 150, 200, 250, 375 and 500 Å thick La_0.7_Sr_0.3_MnO_3_ films grown on (001) oriented LaAlO_3_. (b) Out-of-plane lattice parameters of the La_0.7_Sr_0.3_MnO_3_ films with various thicknesses. *ϕ*-Scans around (101) of the (c) 200 Å and (d) 500 Å thick La_0.7_Sr_0.3_MnO_3_ film. (e) Reciprocal-space-mapping around {101} of the 500 Å thick La_0.7_Sr_0.3_MnO_3_ film. The X-ray diffraction pattern of the 100 Å thick LSMO shows the holder peaks at 44.3°.

The highly aligned grains and in-plane epitaxy were confirmed by asymmetric (101) *ϕ*-scans of LSMO on LAO. The *ϕ*-scans of LSMO show four 90°-separated film peaks [Fig. 1c and d] and are well-aligned with that of the substrate peaks. The *ϕ*-scans of the film and substrate show four-fold symmetry with the cube-on-cube epitaxial growth of LSMO on the LAO substrate. The 150 Å thick LSMO film in the *ϕ*-scan showed relatively broader peaks with FWHM ∼ 1° compared to the 500 Å thick films due to the presence of in-plane misorientation of the pseudocubic unit cells.

Fig. 1e shows the RSMs constructed from the Bragg's reflection measurements consisting of 2*θ*–*ω* coupling scans for different *ω* values of {101} for the 500 Å thick LSMO film grown on LAO. The diffraction peaks associated with the substrate and the film are located at different values of *q*_‖_, indicating that the films are relaxed. In addition, the transverse scattering vectors corresponding to the diffraction peak positions of the substrate and film are different. The angular positions of the Bragg's peaks due to diffraction along 〈101〉 of {101} of LSMO are very close to each other, which confirm the presence of the same in-plane lattice parameters irrespective of 〈101〉. The *c*_pc_ extracted from the RSM is very close to that measured from the symmetric scan. The in-plane lattice parameter of LSMO is *a*_pc_ = *b*_pc_ = 3.87 Å. As the LSMO film thickness decreased below 500 Å, the in-plane lattice parameter remained the same up to 200 Å and then decreased to 3.86 Å for the 150 Å thick film. However, the (101) peak intensity of the 100 Å thick LSMO films was not noticeable to calculate the in-plane lattice parameter. Overall the in-plane lattice parameter of LSMO is close to the pseudocubic lattice parameter of the bulk LSMO with a negligible strain of ∼0.2% [Fig. S2†]. The substrate-induced in-plane compressive strain relaxed to ∼0.2%, while the out-of-plane tensile strain remained at around −2.8% [Fig. S2†] in the LSMO films grown on the (001) oriented LAO substrate. The presence of anisotropic strain may be due to the substrate-induced strain, twin boundaries, and oxygen vacancies.

The bulk LSMO possesses a rhombohedral unit cell with the space group *R*3̄*C* and lattice constants 5.471 Å and *α*_r_ = 60.43°.^17^ In this unit cell, the octahedron rotations can be described by Glazer's tilt system *a*^−^*b*^−^*c*^−^, which consists of equivalent out-of-phase rotations (see ESI†) about the 〈100〉 cubic axes.^18^ Thus, a rhombohedral crystal structure for the LSMO thin films on LAO is expected. However, the X-ray diffraction studies of the LSMO films on LAO in Fig. 1e indicate that LSMO stabilizes in tetragonal structures with the lattice parameters *a* = 5.47 Å (*i.e.*, *a*_pc_ = 3.87 Å) and *c* = 7.98 Å (*i.e.*, *c*_pc_ = 3.99 Å). Thus, the tetragonal structure has the space group *I*4/*mcm* with Glazer's tilt system *a*^0^*b*^0^*c*^−^.^19^ The synthesis of the LSMO film on LAO introduced structural reconstruction of LSMO from *a*^−^*b*^−^*c*^−^ type octahedral tilt to *a*^0^*b*^0^*c*^−^ similar to PrMnO_3_/SrRuO_3_.^20^ Interestingly, the tetragonal LSMO thin film does not conserve the unit cell volume, in contrast to the LSMO film on LAO with a conserved volume.^21^

As shown in Fig. 2, the zero-field-cooled (ZFC) magnetization of the 200 Å thick LSMO film is higher with a relatively sharper peak compared to that of the 500 Å thick LSMO film. The ZFC *M*(*T*) indicates the presence of randomly oriented non-collinear Mn ion spins in the LSMO film. The difference in the ZFC magnetization of these two LSMO films suggests that the 500 Å thick film has comparatively more non-collinear Mn ion spin domains with a coupling energy stronger than the Zeeman energy at 0.01 T field. The difference in ZFC and field-cooled (FC) magnetization at a temperature below the irreversible temperature and the decrease in peak temperature in the ZFC curve with an increase in applied field observed for these LSMO films [Fig. S3†] are the characteristics of the spin-glass state. The 0.01 T FC *M*(*T*) of the 200 Å thick LSMO indicates that the paramagnetic phase transforms into a ferromagnetic phase at a temperature of *T*_C_ ≈ 196 K, which is the *T*_C_ of LSMO. The monotonic increase in the 0.01 T FC magnetization at a temperature below 112 K indicates the existence of uniform exchange coupling of the non-collinear Mn ion spin domains. The distinct change in 0.01 T FC magnetization with the temperature of around 112 K was suppressed after increasing the cooling field to 0.5 T [Fig. S3†] due to the increase in the ferromagnetic domain. The *T*_C_ of the LSMO films decreased from 262 K to 102 K as the film thickness decreased from 500 Å to 100 Å [Fig. S3†]. The increase in the 0.01 T FC magnetization of the 500 Å thick LSMO film at a temperature below 60 K suggests the existence of non-uniform exchange coupling of the non-collinear Mn ion spin domains with ferromagnetic Mn ion spin domains. The *T*_C_ values of the LSMO films are significantly lower than that of the bulk LSMO. However, the observed *T*_C_ values of these films are very close to that of the reported LSMO/LAO.^22^ The *T*_C_ of the LSMO films depends on the spin orientation of Mn, which is determined by MnO_6_. As seen in Fig. 1b, MnO_6_ in the LSMO film on LAO is elongated along the ‘*c*’ direction compared to its bulk. Thus, the reduced *T*_C_ of the LSMO film on LAO compared to its bulk is attributed to possible sources of MnO_6_ rotation and tilting near the interfaces such as surface twining, substrate-induced strain,^8^ and oxygen non-stoichiometry.^23^ The FC *M*(*T*) of the LSMO films with a thickness of <200 Å did not show a clear paramagnetic to ferromagnetic transition, suggesting the richness of the non-collinear Mn ion spin domains [Fig. S3†].

**Fig. 2:**
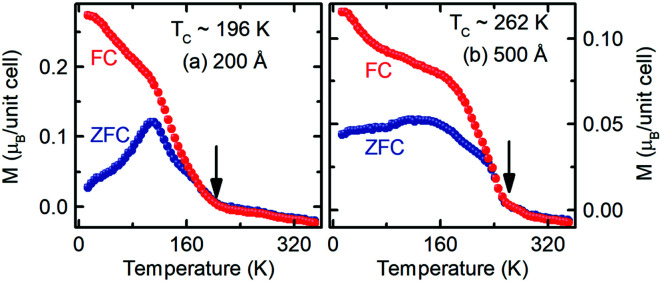
Temperature dependence of the out-of-plane zero-field-cooled and field-cooled magnetization of the 200 and 500 Å thick La_0.7_Sr_0.3_MnO_3_ films grown on (001) oriented LaAlO_3_ in the presence of a 0.01 T field.

The temperature-dependent resistivity (*ρ*(*T*)) of the ∼2 mm wide LSMO films was measured using the two-probe method [Fig. 3a]. The resistance of the 100 Å thick LSMO film at room temperature was ∼170 kΩ, which on cooling below room temperature increased monotonically and became outside the measurable range of the voltmeter at ∼140 K [Fig. 3b]. Qualitatively, a similar *ρ*(*T*) was observed with an extended measurable temperature range after the application of a magnetic field along the out-of-plane direction of the 100 Å thick LSMO film. The (*ρ*(*T*,*H*)) of the 150 Å thick LSMO film [Fig. 3b] is similar to that of the 100 Å thick LSMO film. The *ρ*(*T*) of the LSMO films with a thickness of ≤150 Å did not exhibit metal-like behavior but semiconductor-like behavior. The *ρ*(*T*) data of these films fits well with1
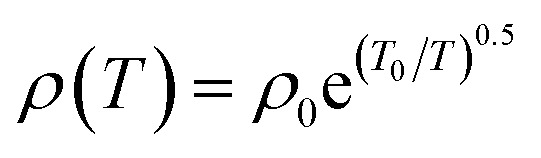
[Fig. 3b and S4†], which indicates that their transport behavior is consistent with the Efros–Shklovskii variable range hopping (ES-VRH) mechanism.^24^ The *ρ*_0_ may be either independent of temperature or a slowly varying function of temperature, while *T*_0_ is a constant of the material, and is related to the rate at which the wavefunction decreases with hopping distance. Thus, the *T*_0_ of the ES-VRH is correlated inversely with the localization length (*ξ*) of the carriers. The *T*_0_ of the 100 Å and 150 Å thick LSMO films obtained from the ES-VRH fit decreased with an increase in the magnetic field [Fig. S4†], which suggests an increase in *ξ* [Fig. 3d]. As the magnetic field increases, the size of the ferromagnetic domain increases, which decreases the spin-dependent scattering; therefore, the increase in *ξ* is expected. The ES-VRH fit suggests the presence of interactions between the localized electrons, which may be due to the structural reconstruction, *i.e.*, the lattice distortion [Fig. 1c] and non-collinear Mn ion spins [Fig. 2].

**Fig. 3:**
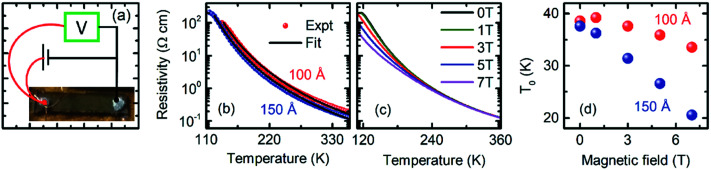
(a) Transport properties measurement configuration. (b) Temperature-dependent zero-field resistivity of the 100 Å, and 150 Å thick La_0.7_Sr_0.3_MnO_3_ films grown on (001) oriented LaAlO_3_. The solid line represents the fit to the Efros–Shklovskii variable range hopping model. (c) Temperature-dependent resistivity of the 150 Å thick La_0.7_Sr_0.3_MnO_3_ films grown on (001) oriented LaAlO_3_ measured in the presence of 0, 1, 3, 5, and 7 T magnetic fields. (d) Magnetic field-dependent *T*_0_ of the 100 Å and 150 Å thick La_0.7_Sr_0.3_MnO_3_ films grown on (001) oriented LaAlO_3_.

The variation in *ρ*(*T*,*H* = 0) of the 150 Å thick LSMO film indicates that the LSMO–LAO interface consists of a semiconducting phase. The semiconducting phase is rich in non-collinear Mn ion spins; thus, around an 80% drop [Fig. 3c] in resistivity in *ρ*(*T*,7*T*) of the 150 Å thick LSMO compared to its *ρ*(*T*,0) at ∼135 K was observed. The large drop in resistivity confirms the significant contribution of the spin-dependent scattering in the absence of a magnetic field.

The resistivity of the 200 Å thick LSMO film on cooling below room temperature increased monotonically and became maximum at a temperature of *T*_P_ ∼ 95 K, which we marked as the metal-insulator transition temperature. Upon further cooling the 200 Å thick LSMO film below *T*_P_, the resistivity decreased gradually and became minimum at a temperature of *T*_Min_ ∼ 45 K, followed by a sharp increase down to the lowest temperature [Fig. 4a]. As *t*_LSMO_ increased above 200 Å, a qualitatively similar *ρ*(*T*,0) was observed with a lower resistivity value, higher *T*_P_, and lower *T*_Min_ [Fig. 4]. The resistivity of the 200 Å thick LSMO film decreased with the application of a magnetic field, which indicates the existence of a negative MR character in the film. As the magnetic field increased, the *T*_P_ of the LSMO film increased, while the *T*_Min_ decreased [Fig. 5a], which is consistent with the formation of a ferromagnetic metallic domain with the magnetic field. As the thickness of the LSMO increased above 200 Å, the resistivity and *T*_Min_ decreased, while the *T*_P_ increased [Fig. 5b]. The increase in the *T*_P_ of the LSMO film grown on LAO with an increase in LSMO film thickness has been reported previously.^25^ The variation in resistivity at temperatures *T*_P_ > *T* > *T*_Min_ can be explained by the double exchange^26^ and phase separation^27^ mechanisms in the LSMO films. The observed variation in resistivity with *t*_LSMO_ is attributed to the expanded ‘*c*_pc_’, which induces a Jahn–Teller distortion along the ‘*c*’ direction and controls the spin reorientations and electron localization.^28^

**Fig. 4:**
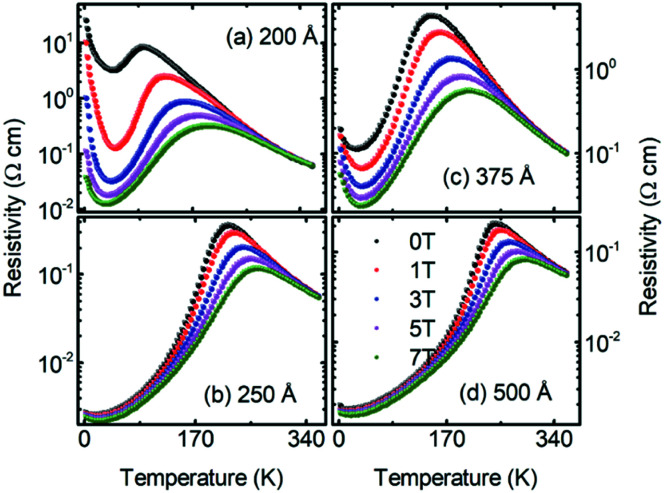
Temperature-dependent resistivity of the of the (a) 200 Å, (b) 250 Å, (c) 375 Å, (d) and 500 Å thick La_0.7_Sr_0.3_MnO_3_ films grown on (001) oriented LaAlO_3_ measured in the presence of 0, 1, 3, 5, and 7 T magnetic fields.

**Fig. 5:**
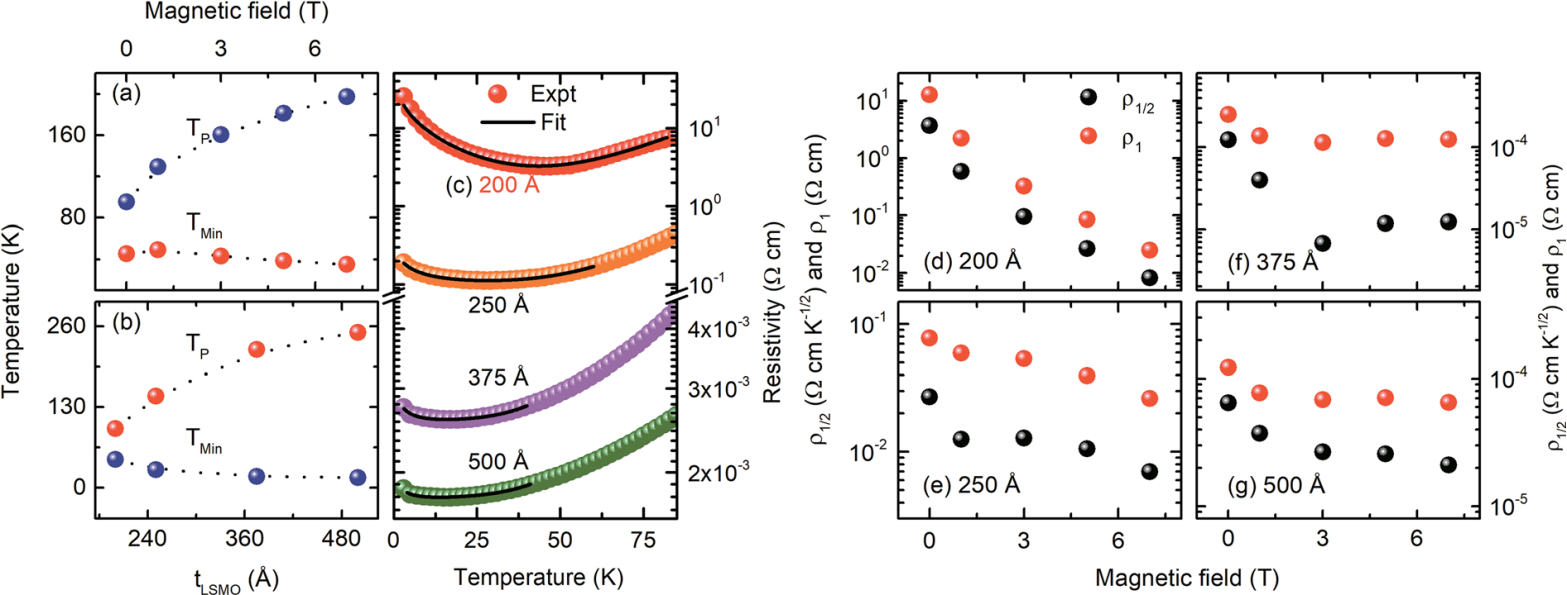
(a) *T*_P_ and *T*_Min_ of the 200 Å thick La_0.7_Sr_0.3_MnO_3_ films grown on (001) oriented LaAlO_3_ as a function of the magnetic field. (b) *T*_P_ and *T*_Min_ as a function of the LSMO film thickness. The dotted line is a guide for the eye. The solid lines in (c) show the fit to the low-temperature resistivity below *T*_Min_ using eqn (3). Magnetic field-dependent fitting parameters *ρ*_1/2_ and *ρ*_1_ of eqn (3) for the (d) 200 Å, (e) 250 Å, (f) 375 Å, and (g) 500 Å thick La_0.7_Sr_0.3_MnO_3_ films grown on (001) oriented LaAlO_3_.

The resistivity at *T* < *T*_Min_ for the LSMO films [Fig. S5†] was fitted using the following expression:^29^2
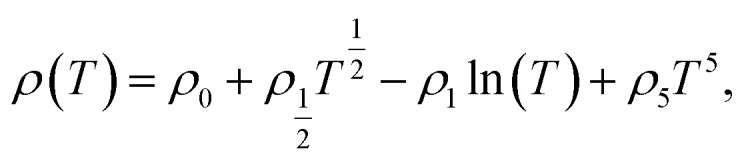
where *ρ*_0_ is the residual resistivity, the second term is due to the contribution of electron–electron scattering, the third term is the spin-dependent Kondo-like effect, and the last term is related to the inelastic scattering. The fitting result as denoted by the solid lines in Fig. 5c [Fig. S5†] agrees well with our experimental data, which indicates that the contribution to the resistivity of LSMO from the inelastic scattering is negligible [Fig. S6†]. As the LSMO film thickness increased, the coefficients *ρ*_½_ and *ρ*_1_ decreased [Fig. 5d–g], suggesting a decrease in electron–electron scattering and spin-dependent Kondo-like scattering, consistent with the observed thickness-dependent *M*(*T*). The coefficients *ρ*_½_ and *ρ*_1_ also decreased with an increase in the magnetic field [Fig. 5d–g]. The coefficient *ρ*_½_ is smaller than the coefficient *ρ*_1_ irrespective of the thickness of the LSMO film or magnetic field value, and thus the electron–electron scattering is smaller compared to the spin-dependent Kondo-like effect. The Kondo effect is attributed to the interaction of conduction electrons with the localized spin at the non-collinear Mn ion spins domain boundary. As the temperature decreases below *T*_Min_, the lattice distortion due to structural reconstruction and magnetic disorder effect becomes dominant, and the resistance upturn emerges.^30^

Fig. 6 shows the temperature-dependent 
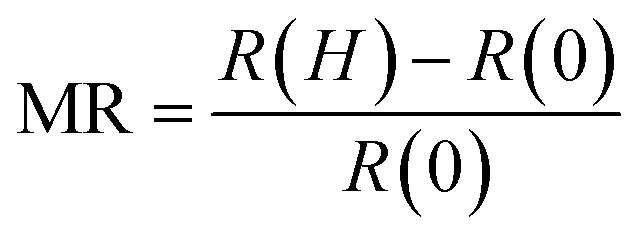
 of the LSMO films with different thicknesses. The MR of the 100 Å thick LSMO film was positive for a 1 T field. As the field increased to 3 T, the MR of this film remained positive at room temperature; however, on cooling below room temperature, the MR decreased and became negative at ∼175 K. A similar temperature-dependent MR (MR(*T*,*H*)) was observed at a higher magnetic field with an enhanced MR and expanded temperature range for the negative MR. At a 1 T field, the MR of the 150 Å thick LSMO was positive in the temperature range of 360 K to 207 K, while it was negative at a temperature lower than 207 K [Fig. 6b]. Upon increasing the magnetic field, although the MR(*T*) curve was similar, the positive MR of the 150 Å thick LSMO decreased, while its negative MR increased. The MR of the 200 Å thick LSMO film was negative at a 1 T field. As the temperature decreased below room temperature, the MR at 1 T increased up to 80 K and remained at ∼96% until 35 K and then started decreasing down to the lowest temperature [Fig. 6c]. Upon increasing the magnetic field, the negative MR of the 200 Å thick LSMO increased, and the plateau became wider. At a 7 T field, the MR of the 200 Å thick LSMO was ∼99% in the temperature range of 3 K to 120 K. As the LSMO film thickness increased, the MR(*T*,*H*) was qualitatively similar to that of the 200 Å thick LSMO with (i) a decrease in MR, (ii) the plateau became a peak, and (iii) the peak became narrow [Fig. 6d–f]. The peak observed in the MR(*T*,*H*) shifted towards a higher temperature with an increase in its sharpness upon increasing the film thickness as the *T*_C_ and ferromagnetic domain size increased [Fig. S3†]. The variation in MR at around 360 K in the MR(*T*,*H*) was negligible since the films were in the paramagnetic state. The constant MR temperature window near 360 K became wider as the film thickness decreased from 500 Å because of the decrease in *T*_C_, *i.e.*, expansion of the paramagnetic state.

**Fig. 6:**
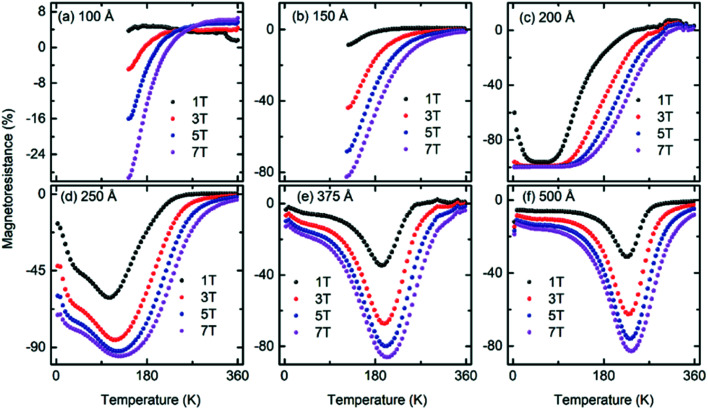
Temperature-dependent magnetoresistance of the (a) 100 Å, (b) 150 Å, (c) 200 Å, (d) 250 Å, (e) 375 Å, and (f) 500 Å thick La_0.7_Sr_0.3_MnO_3_ films grown on (001) oriented LaAlO_3_ measured in the presence of 1, 3, 5, and 7 T magnetic fields.

Fig. 7a shows the isothermal MR(*H*) of the 200 Å thick LSMO film. After applying a current, the voltage was measured as a function of the magnetic field at 10 K from 0 T to 7 T, which is the virgin curve. The virgin curve of the 200 Å thick LSMO, as the magnetic field increased from zero to 7 T, shows a colossal drop in resistance to 99.8%. The MR(*H*) of this film exhibited several unique features such as (i) sharp drop in MR up to 60% under a 300 mT field in the virgin curve, (ii) after decreasing the field from 7 T, the MR remained ∼99% for the entire range of field variation of ±7 T, and (iii) the butterfly shape MR loop is asymmetric [inset of Fig. 7a]. A qualitatively similar MR(*H*) with a lower value of MR was observed at 10 K for the LSMO films of higher thicknesses [Fig. 7b–d]. The variation in the MR at a 300 mT field during the first field increasing branch, and after decreasing the field from 7 T, with the LSMO film thickness, is shown in the inset of Fig. 7e. The asymmetry in the MR(*H*) curve may be due to the exchange biasing between the Mn ion non-collinear spin and ferromagnetic spin in LSMO. The sharp drop in the MR with a low field (<300 mT) indicates that a fraction of the film has non-collinear Mn ion spins with weak magnetic coupling. The gradual drop in MR with an increase in the magnetic field above 300 mT demonstrates the presence of weak magnetic coupling between the Mn ions. The negligible variation in MR after applying ±7 T even at a low field attests the achievement of complete ferromagnetic ordering in the LSMO films. The virgin curve in the MR(*H*) of the 200 Å thick LSMO film at a 300 mT field shows MR 60%, which decreases to 0.7% for the 500 Å thick LSMO film. Similarly, at 7 T field, the 99.8% MR in the MR(*H*) of the 200 Å thick LSMO film decreased to 7.69% for the 500 Å thick LSMO film [Fig. 7]. The observed decrease in MR at 10 K with *t*_LSMO_ is attributed to the decrease in the non-collinear Mn ion spins.

**Fig. 7:**
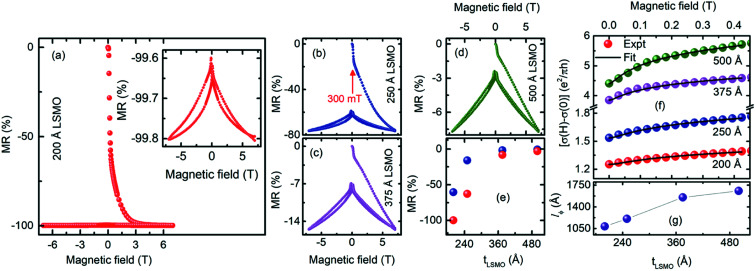
Field-dependent magnetoresistance of the (a) 200 Å, (b) 250 Å, (c) 375 Å, and (d) 500 Å thick La_0.7_Sr_0.3_MnO_3_ films grown on (001) oriented LaAlO_3_ measured at 10 K. Inset of (a) shows the field increasing and decreasing branches of magnetoresistance of the 200 Å thick La_0.7_Sr_0.3_MnO_3_ films measured between ±7 T. (e) Magnetoresistance at 300 mT with an increase in the field from 0 to 7 T (blue sphere) and decrease from +7 T to −7 T (red sphere). (f) Field-dependent change in magnetoconductance and the HLN fit. (g) Variation in the phase coherence length with LSMO film thickness.

The change in magnetoconductance (MC) at a field below 0.4 T for the LSMO films was fitted using the Hikami–Larkin–Nagaoka (HLN) theory [Fig. 7f]. According to the HLN theory, the change in MC in the two-dimensions (2D) is:^31^3
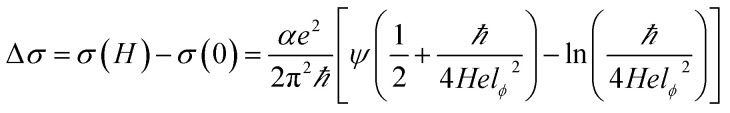
where *ψ* is the digamma function and *α* is a coefficient reflecting the strength of the spin–orbit coupling and magnetic scattering. The value of *α* is 1 if the spin–orbit interaction and the magnetic scattering are weak, *α* = 0 (unitary) for strong magnetic scattering, and *α* = −0.5 for the symplectic case. The *α* value obtained from the fit of Δ*σ*(*H*) for the 200 Å thick LSMO film is 0.08, which increased to 0.54 for the 500 Å thick LSMO. Thus, the LSMO films exhibit weak localization, and the magnetic scattering decreases with an increase in thickness, consistent with the observed reduction in MR with an increase in thickness. The phase coherence length (*l*_*ϕ*_) describes the quantum correction to the conductivity in 2D systems. The *l*_*ϕ*_ obtained from the fit of Δ*σ*(*H*) of the different LSMO films is plotted in Fig. 7g. The *l*_*ϕ*_ values of the LSMO films are larger than the film thickness, which indicates that the charge carriers are confined in 2D. In addition, these *l*_*ϕ*_ values are consistent with that for the previously reported LSMO films grown on SrTiO_3_.^32^ The increase in *l*_*ϕ*_ with the LSMO film thickness is consistent with the formation of ferromagnetic and conducting domains with an increase in the LSMO film thickness on LAO.

According to Hund's rule, the e_g_^1^ electron of Mn^3+^ in manganites couples ferromagnetically to the local spin of the t_2g_^3^ orbital.^1^ However, the thermal energy induces the spin reorientation of the e_g_^1^ electrons, which increases the magnetic disorder and requires a high magnetic field to reduce the spin-dependent scattering, *i.e.*, colossal magnetoresistance.^33,34^ The spin-dependent scattering, *i.e.*, the resistivity of LSMO, was reduced to a significant fraction by the application of *a* < 1000 mT magnetic field, which is associated with a large number of grain boundaries having a non-collinear Mn ion spin structure.^35,36^ Thus, the low field MR of the LSMO films can be attributed to the non-collinear spins of Mn near the LSMO–LAO interface. Further, the observed LFMR in LSMO/LAO is considerably larger than that in the ferromagnetic manganite/spacer/ferromagnetic manganite trilayers, which consist of artificial grain boundaries at the interface.^11–13^ The strain analysis indicates that the effect of strain on the LSMO films with a thickness of 100 to 500 Å is the same. However, the variation in *M*(*T*), *ρ*(*T*,*H*), and MR(*T*,*H*) with film thickness confirms that the orientation of the Mn ion spin plays a vital role in the physical properties of these films. The spin state of the Mn ions in LSMO on LAO is controlled by MnO_6_, which in general can be influenced by the twin boundaries, strain, and oxygen vacancies.

In conclusion, a series of La_0.7_Sr_0.3_MnO_3_ thin films of different thicknesses were grown on (001) oriented LaAlO_3_ by adopting the pulsed plasma deposition method in a sputtering system. The reciprocal space mapping indicated that the in-plane growth of LSMO is relaxed; however, the symmetric XRD scan confirmed the strained growth along the out-of-plane direction with tetragonal structure. The consequences of the LSMO–LAO interface effect appear in the *T*_C_, temperature-dependent resistivity, metal-to-semiconductor transition temperature, and MR. At a magnetic field of 7 T, the MR decreased from 99.8% to 7.69% as the LSMO thickness increased from 200 Å to 500 Å. A unique characteristic of the LSMO films is the large low-field MR after decreasing the field from the maximum field. The observed ZFC and FC *M*(*T*) of the LSMO thin films grown on LAO provide direct evidence that the low field MR in LSMO films on LAO is associated with a large number of grain boundaries having non-collinear Mn ion spins. Thus, the present work demonstrates the great potential of interface and large low-field MR, which may advance the fundamental applications of orbital physics and spintronics.

## Conflicts of interest

The authors declare no competing financial interest.

## Supplementary Material

NA-002-D0NA00287A-s001
