# Supplementary material for: Interfacial reconstruction in La0.7Sr0.3MnO3 thin films: giant low-field magnetoresistance
Source: Nanoscale Adv. 2020 May 7;2(7):2792–9. doi: 10.1039/d0na00287a (PMC9418895; doi:10.1039/d0na00287a)
Supplement: NA-002-D0NA00287A-s001 [file NA-002-D0NA00287A-s001.pdf]

Electronic Supplementary Material (ESI) for Nanoscale Advances.
This journal is © The Royal Society of Chemistry 2020

Supporting Information

Interfacial reconstruction in the $La_{0.7}Sr_{0.3}MnO_3$ thin films: giant low-field magnetoresistance.

Umesh Kumar Sinha, Bibekananda Das, and Prahallad Padhan

Department of Physics, Indian Institute of Technology Madras, Chennai 600036, India

AUTHOR INFORMATION :

Corresponding Author :

Padhan@iitm.ac.in

The substrate $LaAlO_3$ (LAO) is cubic at temperature > 800 °C with space group $Pm\bar{3}m$. [1] On cooling the substrate below 800 °C, the crystals structure of the LAO becomes rhombohedral with the space group $R\bar{3}C$. [2] The structure of LAO at room temperature provides a simple example of octahedral tilting. The AlO_6 tilting can be described by using Glazer [3] symbols of the form $a^{\#}b^{\#}c^{\#}$ in which the literals refer in turn to tilts around axes in the x, y and z directions of the $Pm\bar{3}m$ parent structure. The repetition of a letter indicates that the tilts about the corresponding axes are equal in magnitude. The superscript # takes the value + or – to indicate that the tilts of successive octahedra along the relevant axis are in the same or opposite sense. The terms in-phase (Fig. S1(a)) tilting to describe the former case (+) and out-of-phase (Fig. S1(b)) tilting for the latter (-). For directions about which there is no octahedral tilting, we show the superscript # as 0. Thus, the LAO at temperature > 800 °C exhibits $a^0b^0c^0$ with lattice parameter 3.81 Å, [1] but transform to $a^-b^-c^-$ with lattice parameter 5.36 Å (i.e., Pseudo-cubic lattice parameter 3.79 Å). [2]

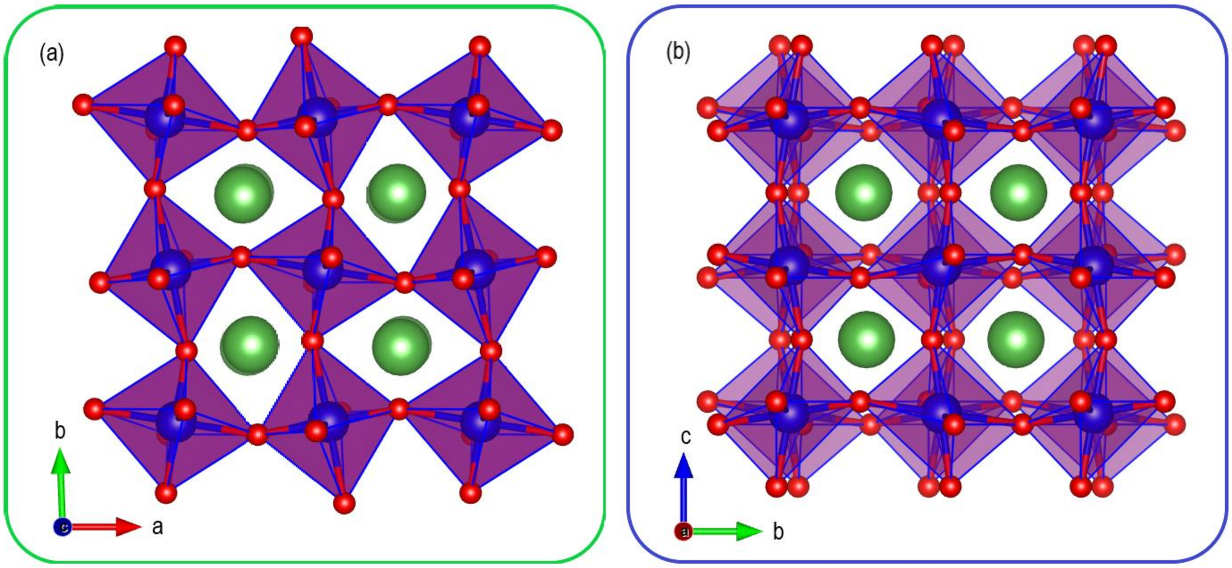

Fig. S1. Schematic for the (a) in-phase and (b) out-of-phase rotation of the MnO_6 in manganites.

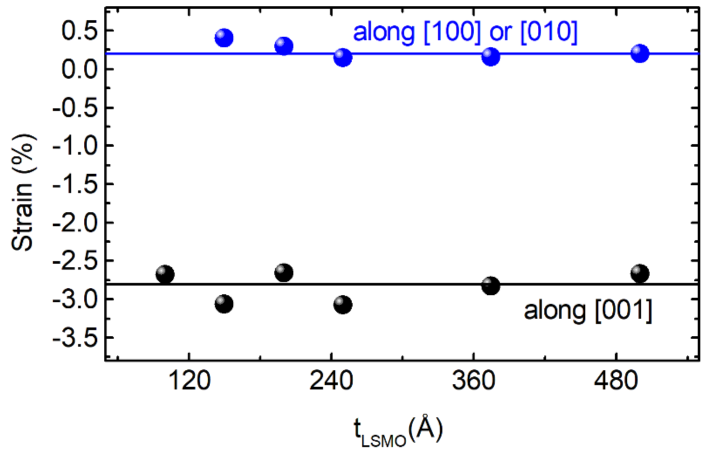

Fig. S2. Strain along the [100] or [010] and [001] of different $La_{0.7}Sr_{0.3}MnO_3$ films grown on (001) oriented $\mathrm{LaAl}O_3$.

The strain on the LSMO thin films grown on (001) $\mathrm{LaAl}O_3$ is calculated using the relations

$$\frac{a_{bulk} - a_{film}}{a_{bulk}} \times 100 \quad or \ \frac{a_{bulk} - c_{film}}{a_{bulk}} \times 100.$$

Fig. S2 shows the in-plane and out-of-plane strain calculated using the (101) and (001) x-ray diffraction scans of the LSMO thin films.

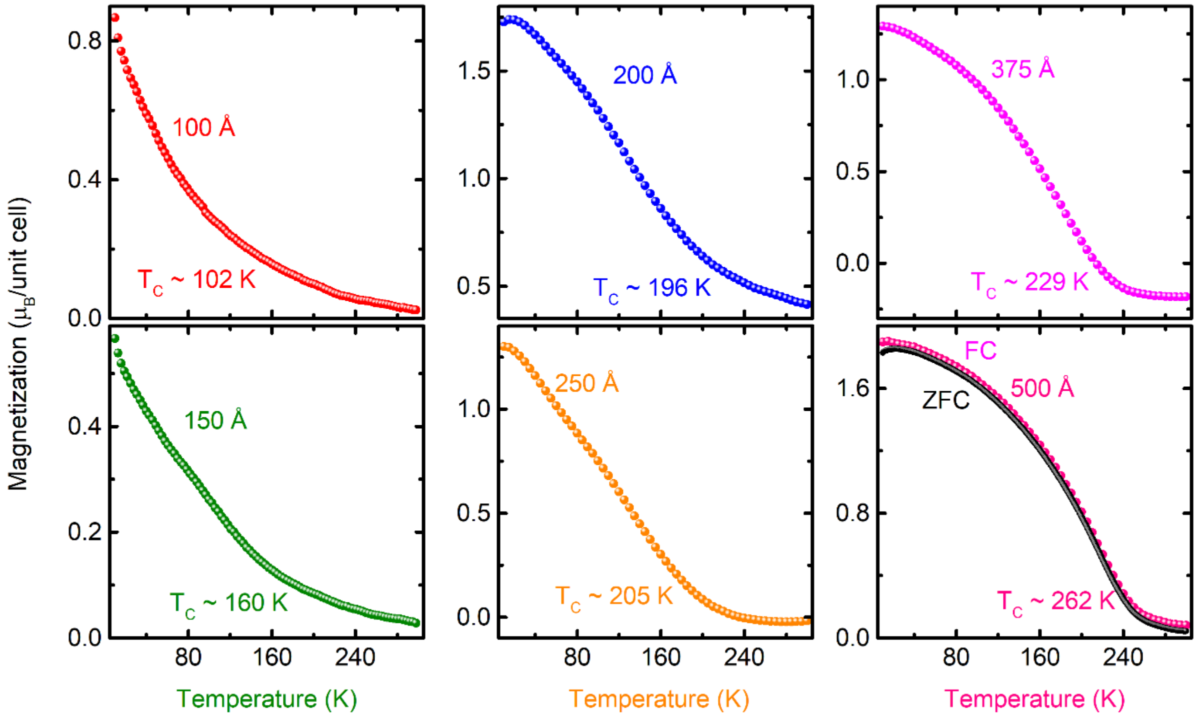

Fig. S3. Temperature-dependent 0.5 T field-cooled magnetization of the 100 Å, 150 Å, 200 Å, 250 Å, 375 Å, and 500 Å thick $La_{0.7}Sr_{0.3}MnO_3$ films grown on (001) oriented $LaAlO_3$. The zero-field-cooled magnetization of the 500 Å thick $La_{0.7}Sr_{0.3}MnO_3$ films is also shown in lower right panel.

Fig. S3 shows the temperature-dependent magnetization of the LSMO thin films with various thicknesses. The Curie temperature of these LSMO films decreases with a decrease in thickness. The T_C of the LSMO films was determined from the intersection of the straight lines, which fit the magnetization on either side of the onset of the ferromagnetic state temperature [4].

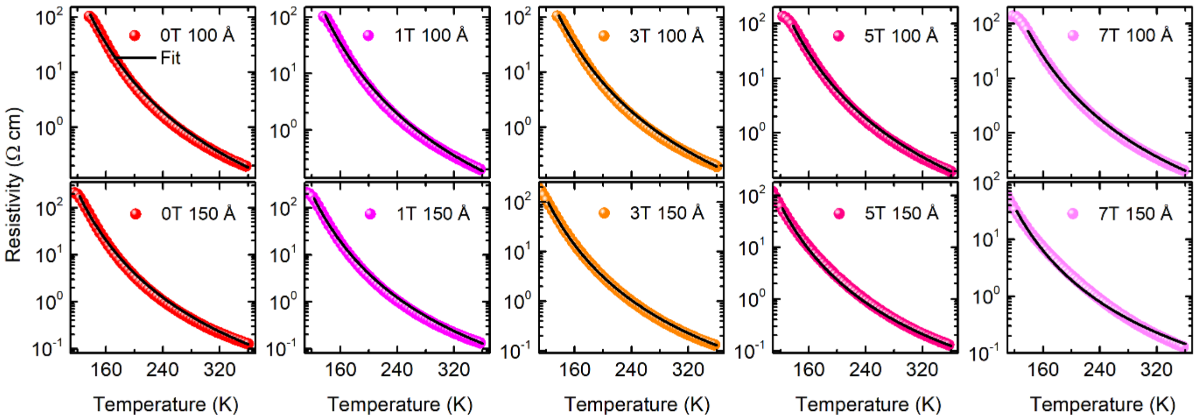

Fig. S4. Temperature-dependent resistivity, measured in the presence of 0, 1, 3, 5, and 7 T magnetic fields, for the 100 Å, and 150 Å thick $La_{0.7}Sr_{0.3}MnO_3$ films grown on (001) oriented $\mathrm{LaAl}O_3$. The solid line represents the fit to the Efros-Shklovskii variable range hopping model.

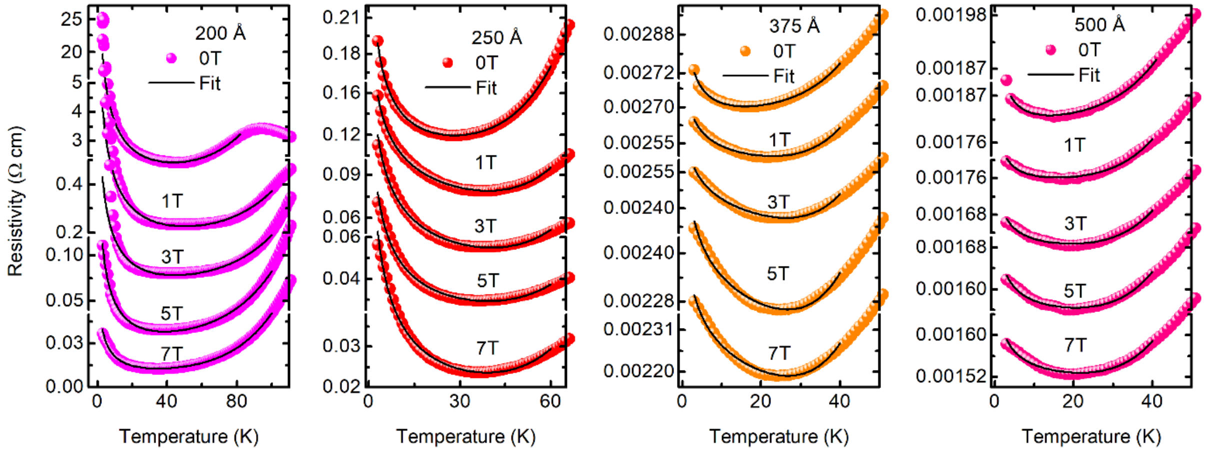

Fig. S5. Temperature-dependent resistivity, measured in the presence of 0, 1, 3, 5, and 7 T magnetic fields, for the 200 Å, 250 Å, 375 Å, and 500 Å thick $La_{0.7}Sr_{0.3}MnO_3$ films grown on (001) oriented $\mathrm{LaAl}O_3$. The solid lines are the fit to the low-temperature resistivity using Eq. 2.

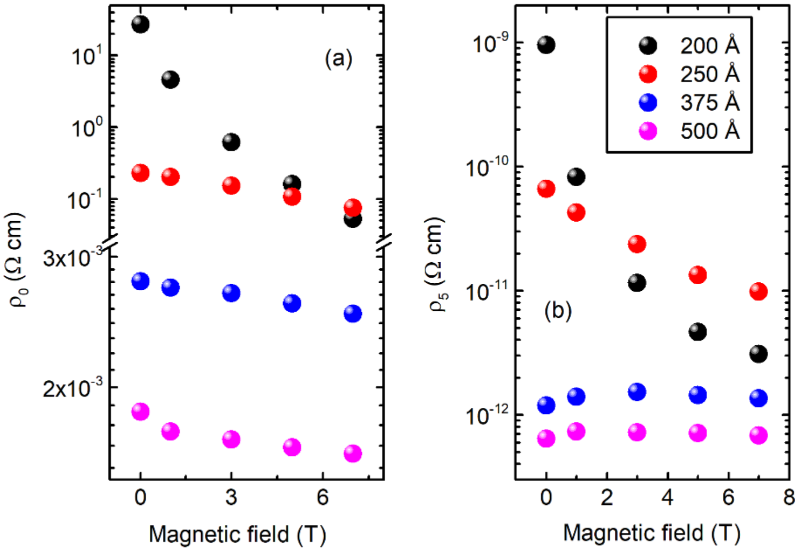

Fig. S6. Magnetic field dependent fitting parameters (a) ρ_0 and (b) ρ_5 of Eq. 2 for the 200 Å, 250 Å, 375 Å, and 500 Å thick $La_{0.7}Sr_{0.3}MnO_3$ films grown on (001) oriented $\mathrm{LaAl}O_3$.

References :

1 S. Geller, and P. M. Raccah, *Phys. Rev. B,* 1970, **2**, 1167.

2 G. W. Berkstresser, A. J. Valentino, and C. D. Brandle, *J. Cryst. Growth,* 1991, **109**, 457.

3 A. M. Glazer, *Acta Cryst. B,* 1972, **28**, 3384.

4 R. López-Ruiz, C. Magén, F. Luis, and J. Bartolomé, *J. Appl. Phys.,* 2012, **112**, 073906.
